# Does Where You Live Matter? An Analysis of Intergenerational Transmission of Education Among Hispanic Americans

**DOI:** 10.3389/fsoc.2021.657980

**Published:** 2021-08-13

**Authors:** Sharron Xuanren Wang, Arthur Sakamoto

**Affiliations:** ^1^ Department of Sociology and Criminal Justice, Delaware State University, Dover, DE, United States; ^2^ Department of Sociology, Texas A&M University, College Station, TX, United States

**Keywords:** Hispanic Americans, immigration, intergenerational educational mobility, county-level characteristics, NLSY97

## Abstract

The intergenerational transmission of education from parents to children is an important indicator of societal inclusiveness and educational inequality. The present study uses restricted-access data from the National Longitudinal Survey of Youth 1997 (NLSY97) to investigate whether intergenerational educational transmission varies by county-level demographic and socioeconomic characteristics for Hispanic Americans. Based on parental birthplace, Hispanic Americans are grouped into 3 + generation (i.e., children of native-born Hispanic parents) and 2nd generation (i.e., children of foreign-born Hispanic parents). Men and women are analyzed separately. The results indicate that intergenerational educational mobility is higher if 3 + generation Hispanic men reside in areas with a larger Hispanic population, and if 2nd generation Hispanic men reside in areas with a larger college-educated population, during their adolescent years. County-level socioeconomic characteristics do not seem to affect intergenerational educational mobility of Hispanic women, non-Hispanic white men, or non-Hispanic white women. Theoretical and empirical implications of the findings are discussed.

## Introduction

Hispanic Americans now constitute the largest minority group in the United States Understanding the sources of their socioeconomic status is important for providing a more accurate appraisal of racial/ethnic inequality. It is reported that on average Hispanic Americans have the lowest educational level among racial/ethnic groups in the U.S. For example, according to the U.S. Census, 58% of Asians have a bachelor’s degree or higher, followed by non-Hispanic Whites (40%) and Blacks (26%). Only 19% of Hispanics have a bachelor’s degree or higher (U.S. Census Bureau 2019). In addition, among Hispanics, foreign-born Hispanics demonstrate a lower educational level than native-born Hispanics (Perlmann, 2005). Twenty-percent of native-born Hispanics have a college degree compared to 12% of foreign-born Hispanics in 2015 (U.S. Census Bureau 2015).

As education has become all but vital for social mobility and long-term economic success (Hout, 2012), low educational attainment has become a barrier to the social and economic advancement of many Hispanic Americans (Perlmann, 2005). In general, education provides immigrant children with the opportunity to advance their economic success as adults as well as a means to foster assimilation (Zhou, 1997; Baum and Ma, 2007; Hirschman, 2016; Wang and Sakamoto, 2021). Previous studies have suggested that educational attainment is heavily influenced by family background, including parental educational level, family economic resources, family structure, number of siblings, as well as parental involvement and styles (Becker and Nigel, 1986; Haveman and Wolfe, 1995). Many studies have also emphasized the importance of neighborhood quality as a factor affecting children’s educational attainments (Borjas and George, 1992; Kremer, 1997; Patacchini and Zenou, 2011).

In regards to the educational attainments of children from immigrant families, prior studies have suggested that children of immigrants usually outperform first generation immigrants and children of native-born Americans (Kao and Tienda, 1995; Fuligni, 1997; Crosnoe, 2013). Net of parental socioeconomic status, children from immigrant families also tend perform better academically than children of US-born parents of the same racial, ethnic, or national background (Crosnoe, 2013). This phenomenon is often termed by social scientists as “immigrant optimism” and as one of the “immigrant paradoxes” (Kao and Tienda, 1995; Feliciano, 2005; Crosnoe, 2013). Evidence also indicates that intergenerational educational mobility is typically high among children of immigrants. Children of Latin American immigrants, however, seem more likely to be of a low socioeconomic status as they demonstrate a low level of upward mobility (Duncan and Trejo, 2011a) and therefore may be an exception to the “immigrant optimism” thesis. Despite the fact that the educational attainment among Hispanics has been rising steadily in recent years (Pew Research Center 2016), Hispanic Americans have lower-than-average educational attainment. This low educational attainment has stalled Hispanic Americans’ socioeconomic advancement. Hispanics thus constitute an important case that needs further elucidation, especially with regard to understanding their parental transmission of educational attainment, immigration status, and community contexts.

Extensive prior research has suggested that educational attainments are not only determined by parental socioeconomic factors, but also by the socioeconomic and demographic characteristics of the places where they were raised (Borjas and George, 1992; Kremer, 1997; Kibria, 2003; Patacchini and Zenou, 2011). Although the magnitude of the effect of neighborhood characteristics on education is debatable (Solon, 1992; Katz et al., 2001), many researchers investigating immigration assimilation suggest that neighborhood quality is especially important for socioeconomic outcomes among children from immigrant families (Lee and Zhou, 2015; Tran, 2016). Social context is an important factor that influences assimilation (Tran, 2016), and contextual socioeconomic characteristics play an important role in immigrant assimilation and upward mobility. Even though many immigrants move to the U.S. with a low socioeconomic status, their children perform well academically. Scholars suggest that it might be the socialization from peers that contribute to their upward educational mobility (Lee and Zhou, 2015; Tran, 2016). Children’s development is not only affected by what’s happening “inside the family” (i.e., parental educational level and involvement), but also what’s happening “outside the family” (i.e., neighborhood quality, peer socialization) (Bisin and Verdier, 2000). Patacchini and Zenou (2011) found that neighborhood quality significantly affects educational attainment of children with low-educated parents. Thus, there might be an interactive relationship between parental education and contextual characteristics that influences native-born Hispanic American’s educational attainment.

Intergenerational transmission of education from parents to children may vary according to the demographic and socioeconomic characteristics of the neighborhoods where the child was raised. There are three different ways in which contextual level characteristics can affect how the educational level of Hispanic American parents is transmitted to their children. First, Hispanic children who grew up in an area with a high level of socioeconomic capital might demonstrate higher educational attainment. If this is the case, then contextual capital would positively affect Hispanic children’s education. Second, contextual level capital might decrease Hispanic children’s educational attainment, especially if a child feels disadvantaged in the community and the environment hinders the child’s development. Lastly, contextual level capital might not influence intergenerational transmission of education among Hispanics.

The present study uses restricted-access data from the National Longitudinal Survey of Youth 1997 (NLSY97) to investigate whether the educational transmission from parents to children varies by county level demographic and socioeconomic characteristics among native-born Hispanics. The Hispanic population demonstrates a high level of heterogeneity in terms of generational status. Each generation has a different socioeconomic circumstance and assimilation level (Wang and Sakamoto, 2021). Therefore, based on parental birthplace, the present study groups Hispanics into children of native-born Hispanic parents (i.e., 3 + generation Hispanics) and children of at least one foreign-born Hispanic parent (i.e., 2nd generation Hispanics). Native-born non-Hispanic whites (i.e., 3 + generation whites) are also included in the study as a reference group. This study contributes to the literature on Hispanics’ educational mobility and assimilation in order to better understand the dynamics of immigrant assimilation, which may lead to better strategies for helping children of immigrants achieve greater success in their educational attainments and labor market outcomes.

## Background

### Intergenerational Transmission of Education and Its Mediators

Educational mobility is a useful measure of intergenerational mobility, considering that it is a key aspect of human capital that affects a person’s overall socioeconomic status (Mazumder, 2018). Intergenerational transmission of education is regarded as one of the central mechanisms underlying educational inequality and immigrant assimilation. One way to measure intergenerational educational mobility is to use parent-child schooling association (Andrade and Thomsen, 2018). A high degree of intergenerational transmission of education indicates that parental educational background plays an essential role in children’s education, whereas a low degree of transmission suggests that adult children’s educational attainment is less affected by their parent’s educational level. Using PSID data, Hertz et al. (2007) reported that the correlation coefficient of intergenerational educational mobility in the U.S. is around 0.46, which is lower than Latin American countries, but higher than Nordic countries (Hertz et al., 2007).

Researchers of economics (Borjas and George, 1992; Hertz et al., 2007; Patacchini and Zenou, 2011) and sociology (Feliciano and Lanuza, 2017; Andrade and Thomsen, 2018) have long been interested in how parental education affects their children’s later educational outcomes. Studies have suggested that highly educated parents make more money and therefore are better able to support their children’s education, equipping them with a high level of human capital (Chiu et al., 2016). In addition, educated parents might have a more effective parenting style than those who are not educated, and therefore children of educated parents achieve higher education (Okpala et al., 2001). Educational theories emphasize the importance of parental human capital. For example, research finds that family background accounts for up to 85% of the explainable variation in children’s school attainment (Belzil and Hansen, 2003). In addition, Woessmann (2004) suggests that the explanatory power of parental background in models of educational outcomes decreases the effects of school and institutional effects. In other words, parental background is more important than school in determining a child’s educational attainment. Therefore, parents’ educational attainment and class status significantly affect the academic performance of their children (Coleman, 1968).

Immigrants who perform well in the first generation also tend to perform well in the second (Feliciano, 2005). For example, children of highly educated immigrant parents consistently perform better in school than the descendants of poorly educated parents (Hirschman and Falcon, 1985; Feliciano, 2005). Research has indicated that parental schooling is the most important factor in explaining educational differences across groups (Hirschman and Falcon, 1985; Feliciano, 2005).

Previous studies have suggested that factors influencing intergenerational transmission of education from parents to children might include family income, family structure, number of siblings, as well as parental involvement and parenting style (Okpala et al., 2001; Chiu et al., 2016; Egalite, 2016). Parental income provides the means for parents to transfer their human capital to their children (Hill and Duncan, 1987). The parental investments perspective emphasizes how the earnings capacity and other resources of parents affect the educational attainments and earnings capacity of children (Solon, 1992), in that the parental generation uses their resources to invest in their children’s education, thus enhancing the earnings capacity of their children (Becker and Nigel, 1986; Kao and Tienda, 1995; Feliciano, 2005). Children from low-income backgrounds are more likely to have lower educational attainments (Duncan et al., 1998) as well as lower household incomes or earnings in adulthood compared to those from high-income households.

Family socioeconomic background and structure also influence the education of children of immigrants (Portes and Hao, 2004). A prior study shows that children living in one-parent or other blended types of families tend to be disadvantaged in terms of socioeconomic status, education, and life chances (Hernandez, 1993). Among immigrants, research has suggested that a large number of children from immigrant families are raised by a single parent (Landale and Oropesa, 1995). This family disruption might limit 2nd generation children’s access to parental investment even if their parents work hard and have a high level of human capital, such as education and income (Landale and Oropesa, 1995).

In addition, number of siblings has an unfavorable effect on one’s educational attainment. Becker and colleagues (1973) have suggested that with given resources, parents can either have many children in which they invest little, or they can have few children allowing for greater investments per child and a higher “quality” upbringing. In other words, parents choose between quality and quantity of children. Nguyen and Getinet, 2003 have found that the presence of more than two siblings has a negative effect on children’s educational attainments in the U.S. Bjorklund and Jantti (1999) have suggested that in Finland, Sweden, and the U.S., children from large families are likely to achieve less socioeconomically than children born in small families.

Previous studies have suggested that parenting behaviors also influence the transmission of education across generations (Capaldi and Clark, 1998; Crosnoe and Ansari, 2016). Students from immigrant families who perform well in school tend to be supported by their family (Fuligni, 1997). Based on measures of warmth and supervision in the parent-child interaction, psychologist Baumrind (1967) categorized four kinds of parenting styles, including authoritative (i.e., parents display high levels of both warmth and supervision), authoritarian (i.e., parents display high supervision but low warmth), permissive (i.e., parents display low supervision but high warmth), and disengaged (i.e., parents display both low warmth and low supervision). Research has indicated that an authoritative parenting style leads to favorable outcomes among their children (Maccoby and Martin, 1983). In contrast, a disengaged parenting style has negative consequences for children.

### The Role of Contextual Level Characteristics: Social Landscape of Counties

Extensive research has emphasized the importance of neighborhood social capital and institutional resources on children’s future outcomes (Sampson et al., 2002). Regarding children from immigrant families, Borjas and George (1992) has coined the term “ethnic capital” to illustrate how children of immigrants are influenced by the community in which they were raised. In his study, Borjas focuses on a set of variables involving ethnic skills of different groups to analyze intergenerational mobility across first generation immigrants and their children (Borjas and George, 1992). He suggests that if a child grows up in a community with a high proportion of immigrants, the child is more likely to associate with immigrants, which may influence the skills, language, and educational attainments of the child (Borjas and George, 1992; Borjas and George1995; Borjas, 2006). Borjas also finds that being raised as part of a low-skilled community may reduce intergenerational mobility (Borjas and George, 1992). However, Borjas (2006) suggests that children’s continual exposure to a particular ethnic norm may pull the child toward that norm in the ethnic group, which may hinder the child’s assimilation into mainstream society. Portes and MacLeod (1996) suggest that, in addition to parents, the strength of communities, including social networks and resources, are crucial factors influencing the attainments of immigrant children. Therefore, family background alone cannot explain the variation in educational outcomes among children from immigrant families (Rumbaut, 1994; Fuligni, 1997).

Several recent studies have emphasized the significance of contextual level characteristics in explaining group socioeconomic differences (Kasinitz, 2008; Tran, 2016). Group-level resources might substantially affect the assimilation of the second generation. Under certain circumstances, ethnic capital could benefit specific immigrant groups. Ogbu (1974), Ogbu (1991), Ogbu (2003) suggests that immigrant minorities might develop a positive view of shared heritage in the community, providing a sense of group pride, which in turn might stimulate the success of the next generation. Taken together, these family and community relations are regarded as “ethnicity as social capital” (Bankston et al., 1997).

For instance, Asian Americans on average have a higher socioeconomic status than the general population (Sakamoto et al., 2009; Kim and Sakamoto, 2010). Second generation Asian immigrants tend to achieve high mobility regardless of their parents’ socioeconomic status in the U.S. For example, Vietnamese and Sikh immigrants have lower socioeconomic attainments after coming to the U.S., whereas their children often excel in school beyond what is expected, considering their background (Hsin and Xie, 2014). Evidence indicates that family and individual background factors cannot always fully explain second generation Asian Americans’ high achievement (Goyette and Xie, 1999; Sakamoto et al., 2009). Some scholars use ethnic capital to help explain the achievements of second-generation Asians Americans. For example, Zhou and Bankston (1998) describe how co-ethnic social support and control promote positive incorporations of second generation Vietnamese into a low-income neighborhood in New Orleans. Lee and Zhou (2015) suggest that Asian American children benefit from the ethnic environment in which they are raised, as they witness the behavior and achievements of their peers who share the same national origin or pan-ethnic background regardless of their parents’ socioeconomic status. In contrast, little is known about the role of contextual level capital in Hispanic Americans’ educational attainments and assimilation.

Previous literature has suggested that both parental and contextual characteristics have important effects on educational attainments among children from immigrant families. The association between the two, however, has not been examined. It is possible that parental education and contextual level characteristics not only have individual effects on children’s educational attainment, but that these two factors also have a compounded association with a child’s educational attainment. Findings of the present study may lead to insightful theoretical implications regarding the interaction between parental and contextual capital resources on assimilation and education of descendants of immigrants. In addition, the present study may have important implications for developing new policies geared toward promoting educational mobility among Hispanic/Latino youths. If county level characteristics is associated with increases in educational mobility, new policies should be put in place for improving neighborhood quality in order to increase upward mobility of disadvantaged Hispanic children.

It is not clear whether and how an interactive relationship between county level human capital and parental education exists. Intergenerational transmission of education from parents to children might not be affected by contextual environment. It may be hypothesized that Hispanic children from an area with a larger ethnic population and a higher level of socioeconomic resources may demonstrate greater intergenerational educational mobility. This would indicate a negative interaction between parental education and contextual variables. In this situation, contextual characteristics play a positive role in educational attainment. In addition, we hypothesize that this interactive relationship between contextual level characteristics and parental education exists only among Hispanic Americans, as this group is more vulnerable to environmental factors than 3 + generation non-Hispanic white Americans.

## Data and Methods

### Data and Sample

The National Longitudinal Survey of Youth 1997 (NLSY97) is an ongoing nationally representative panel study of 8,984 youths aged 12–17 when first interviewed on December 31, 1996. The NLSY97 consists of a set of comprehensive surveys providing information about educational and labor market activities at multiple time points. The sample consists of a nationally representative sample of 6,748 youths and an over-sample of 2,236 Hispanic and non-Hispanic black youths. NLSY97 is designed to document the transition from school to work, and thus it collects detailed information about educational experiences over time among young adults. The content of the survey includes detailed information about youths’ educational data and family background. Sources of data include the youths’ parents and the youths themselves. In this study, I use surveys from Round 1 (1997), and from Round 11 (2007) to Round 16 (2013). Many variables in the study are derived from the youth questionnaires. Information on parental and household background is extracted from the Round 1 parent survey. It should be noted that over 80% of the parent surveys were answered by the biological mother of the youth.

Access to the NLSY97 Geocode data file is restricted from the public. It contains detailed information on county-level contextual characteristics of each NLSY resident. The variables indicating county-level demographic and socioeconomic characteristics in the Geocode data files are obtained based on the 1994 edition of the U.S. Census Bureau’s *County and City Data Book*. Therefore, the restricted data contains important contextual level variables useful for the present study.

### Target Population, Variables, and Measures

The target population of this study is Hispanic Americans. Hispanic Americans are grouped into children of native-born Hispanic parents (i.e., 3 + generation Hispanics) and children of at least one foreign-born parent (i.e., 2nd generation Hispanics). The parent survey provides information on parent’s place of birth. Hispanic youth is coded as 2nd generation if at least one of the parents is foreign-born. Hispanic youth is coded as 3 + generation if both parents were born in the U.S. My sample yields 983 Hispanics from native-born families (i.e., 3 + generation Hispanics: N = 983) and 917 Hispanics from immigrant families (i.e., 2nd generation Hispanics: N = 917). It should be noted that we grouped 1.5 generation Hispanics into 2nd generation Hispanics. 1.5 generation Americans are persons who were born outside the U.S. but migrated to the U.S. as a minor. Previous immigration studies have suggested that 1.5 and 2nd generation Americans have similar upbringing and present a similar level of assimilation in the U.S. (Rumbaut, 1994). Thus, conventional studies often group 1.5 generation persons and 2nd generation into one group of children from immigrant families. In addition, I include children of native-born non-Hispanic whites (3 + generation whites) as a reference group (N = 4,184).

*Dependent Variable.* The dependent variable of interest is years of schooling by age 28. It is a continuous variable. Age 28 is used to determine education level, because by 28 most people should already have finished their education, including persons working on a PhD or a professional degree. Using years of education to measure educational attainment also has several advantages, including its wide availability, unambiguity, and remaining stable and fixed after early adulthood (Hertz et al., 2007). I use surveys from Round 11 to Round 16 (from years 2007–2013) to extract years of schooling at age 28 for respondents. At Round 16, the youngest respondents in the sample have reached age 28.
*Key Independent Variables*. The key independent variables include father and mother’s years of schooling. In the parent survey, the responding parents were asked to provide information about their years of schooling, as well as information about youth’s other biological parent, and residential father/mother. If biological parent’s information on education is missing, we use residential parent’s years of schooling.
*Contextual Variables.* We use four demographic and socioeconomic variables from the restricted-access data file as proxies of contextual quality. All these variables are measured at the county level in 1997. These include percentage of ethnic population, percentage of college educated population, natural logarithm of median household income adjusted to 2010 dollars based on CPI-R-U, and percentage of families below poverty level. It should be noted that for Hispanics, ethnic population is obtained by using Hispanic population in county divided by total population in county; and for non-Hispanic whites, ethnic population is obtained by using non-Hispanic white population in county divided by total population in county. All contextual level variables are treated as continuous variables.
*Control Variables.* We use factors influencing intergenerational transmission of education as control variables. Natural logarithm of annual household income in 1996 is included. We adjusted parental household income based on CPI-R-U to 2010 dollars. Annual household income below $1,000 is recoded to $1,000 to avoid biases produced by outliers. We have also included number of siblings in the household and mother’s age when she had the child. These variables are treated as continuous variables. Furthermore, we have included variables indicating whether the child lived with both biological parents to represent family structure, in addition to whether the parents participated in teacher-parent meetings and whether parents volunteered in schools as proxies for parental involvement. These variables are coded as dichotomous variables (yes = 1/no = 0). In addition, based on Baumrind’s parenting styles on warmth and supervision in the parent-child interaction, four dichotomous variables of parenting styles (authoritative, authoritarian, permissive, and disengaged) are included (yes = 1/no = 0). We also have two dichotomous variables indicating region, including metropolitan area and South in the study, as research has suggested that region of residence could affect one’s later educational attainment (Scott, 2009). For example, children from the South are more likely to have a lower educational attainment than children who grew up in the North. In addition, children who are brought up in metropolitan areas are more likely to have a higher level of education than children from rural areas. Lastly, we include a set of variables indicating school quality when youths were teenagers. Studies suggest that school quality is one of the important factors affecting one’s educational outcomes. This is especially true for immigrant families where parents have limited U.S.- specific knowledge to help with their children’s schooling (Crosnoe, 2004). These school quality variables include whether they feel safe at school, whether teachers are engaged, and whether students disrupt the learning process (yes = 1/no = 0).

It is worth noting that there are many missing values in the dataset due to the nature of panel surveys. For the dependent variable of education, we use listwise deletion to deal with missing values. For the independent variables of parental schooling and family income, multiple imputation is used (Royston, 2004).

### Methods

As a standard intergenerational mobility model, we apply the OLS regression model for estimating correlations between educational attainments of parents and children (Solon, 1992; Zimmerman, 1992):

$$Y=\alpha_{1}+\beta_{1}*PE+\beta_{2}*CC+\beta_{3}*X+\epsilon$$ (1)

$$Y=\alpha_{2}+\beta_{4}*PE+\beta_{5}*CC+\beta_{6}*\left(PE*CC\right)+\beta_{7}*X+\varepsilon_{i}$$ (2)

where Y indicates child’s years of schooling; PE indicates parent’s education (i.e., father’s years of education and mother’s years of education); CC indicates a vector of county-level characteristics (i.e., percentage of ethnic population, percentage of college-educated population, natural logarithm of median household income, and percentage of families below poverty level); X indicates a vector of control variables (i.e., natural logarithm of annual family income, family structure, number of siblings, parenting styles, region, and quality of school); and ε indicates random errors. The intergenerational transmissions of father’s education and of mother’s education are analyzed separately. In addition, each of the county-level demographic and socioeconomic characteristics (percentage of ethnic population, percentage of college-educated population, natural logarithm of median household income, and percentage of families below poverty level) are also analyzed separately in the models.

Prior literature has suggested that girls and boys of immigrant backgrounds undergo different assimilation processes and educational outcomes due to differences in institutional barriers (Gillborn and Mirza, 2000; Lopez, 2003). Therefore, all the analyses are conducted separately for men and women. The analyses are weighted using sample weights. The Bureau of Labor Statistics also generated custom weights for multiple samples based upon requests. In model 1, $\beta_{1}$ represents the parent-child school correlation coefficient net of the controls. The larger the value of $\beta_{1}$, the lower the educational mobility and the greater the child educational outcomes depending on parental education. In model 2, the coefficient of the interaction terms ($\beta_{6}$) is the parameter of interest because a significant estimate of $\beta_{6}$ indicates significant heterogeneity in intergenerational mobility based on level of county-level characteristics. This approach is consistent with prior research on intergenerational mobility (Huang, 2013).^1^ If $\beta_{6}$ is statistically not significant, it indicates intergenerational transmission of education from parents to children is not affected by contextual environment. A positive sign of $\beta_{6}$ indicates that county level demographics or socioeconomic characteristics increase intergenerational transmission of education and a negative sign indicates the opposite.

## Results

### Sample Characteristics

Sample characteristics by race, immigration generation, and gender are reported in Table 1. Child’s educational attainment is measured at age 28 for all respondents. In general, women have acquired more years of schooling by age 28 than men. Native non-Hispanic whites have more years of schooling than Hispanics. On average, NH-white women have the highest educational attainment (14.26 years) among all the demographic groups. 2nd generation Hispanic men and women have more years of schooling than 3 + generation Hispanic men and women, respectively. 3 + generation Hispanic men have the lowest educational level among all the groups. The results are consistent with prior findings indicating that 3 + generation Hispanic Americans have shown downward educational mobility (Duncan and Trejo, 2011b; Terriquez, 2014).

**TABLE 1:** Sample descriptive statistics.

	NH-Whites_3+		Hispanics_3+		Hispanic_2nd	
	Men	Women	Men	Women	Men	Women
**Dependent Variable**						
Average years of Schooling by age 28	13.60	14.26	12.53	12.93	12.58	13.04
**Parental and Family Variables (1997)**						
Average father’s schooling (Years)	13.39	13.31	11.02	11.24	8.73	9.00
Average mother’s schooling (Years)	13.30	13.28	11.24	11.18	9.06	8.98
Average in-family income	11.05	11.03	10.42	10.43	10.15	10.17
Average mother’s age when child was born	26.14	25.96	24.70	25.27	25.75	25.34
Average no. of siblings	1.24	1.25	1.61	1.54	1.81	1.89
Lived with both parents (%)	59.13	56.65	45.63	43.98	64.00	59.31
Parenting styles (%)						
Authoritative	41.95	38.63	42.72	38.72	44.70	39.35
Authoritarian	10.58	12.74	9.39	13.27	12.19	15.05
Permissive	37.45	37.83	36.21	34.07	33.18	31.40
Disengaged	10.02	10.80	11.69	13.94	9.93	14.19
(Total)	(100.00)	(100.00)	(100.00)	(100.00)	(100.00)	(100.00)
Parents volunteered in school (%)	49.26	48.91	31.18	32.82	33.11	30.62
Participate Teacher-Parent meetings (%)	59.73	58.23	44.49	44.2	70.89	69.16
**School Environment (1997)**						
Feeling safe at school (%)	88.93	89.53	87.26	85.56	86.44	82.44
Engaging teachers (%)	86.62	85.86	88.78	85.34	88.89	86.72
Students disrupt learning (%)	58.58	60.91	62.74	63.46	65.78	61.67
**Geographical Variables (1997)**						
Metro (%)	75.42	75.35	91.83	91.68	98.89	97.86
South (%)	28.74	32.69	31.94	27.79	28.67	25.91
**County-level Characteristics (1997)**						
% Ethnic Group	86.02	85.86	27.82	26.15	27.33	26.97
% College Educated	19.09	19.06	20.84	20.95	22.1	21.99
Median Household Income ($)	35,562.98	35,540.76	34,579.48	35,456.45	37,617.21	37,181.53
% Family below poverty	9.16	9.18	13.17	12.14	11.79	11.94
N	2,168	2,016	526	457	450	467

Compared to parents’ schooling, children’s schooling is higher among all groups than their father and mother’s schooling, indicating an intergenerational improvement in education among all groups. Overall, parental socioeconomics characteristics of NH-whites are higher than Hispanics (i.e., parents’ schooling and family income).

### Parent-Child Correlation of Schooling

Table 2 presents father-child and mother-child correlation coefficients of schooling across demographic groups while controlling for the covariates including natural logarithm of family variables, school variables, geographic variables, and ethnic capital variables. All the coefficients of parents’ schooling on child’s schooling are statistically significant at a *p* < 0.001 level, indicating a positive association between child’s and parent’s schooling when other observed factors are controlled for. Across all demographic groups in our study, children have reached a higher level of educational attainment than their parents. Specifically, the overall correlation coefficients of parent-child schooling among NH-whites ranges from 0.23 to 0.33 (father-son: 0.31; father-daughter: 0.23; mother-son: 0.28; mother-daughter: 0.33). In addition, the correlation coefficients for second generation Hispanics range from 0.17 to 0.23 (father-son: 0.23; father-daughter: 0.18; mother-son: 0.21; mother-daughter: 0.18), while the correlation coefficients for 3 + generation Hispanics range from 0.19 to 0.27 (father-son: 0.22; father-daughter: 0.20; mother-son: 0.27; mother-daughter: 0.19).

**TABLE 2:** Correlation coefficients of parent-child schooling.

	NH-Whites_3+		Hispanics_3+		Hispanic_2nd	
	Men (1)	Women (2)	Men (3)	Women (4)	Men (5)	Women (6)
**Panel A: Father’s Schooling**	0.3080***	0.2279***	0.2221***	0.2011***	0.2275***	0.1782***
Controls	Y	Y	Y	Y	Y	Y
Intercept	−27.9450***	−18.7090*	−37.4043+	−22.2189	16.3772	−2.5334
R2	0.3234	0.2793	0.2343	0.2627	0.2482	0.2644
**Panel B: Mother’s Schooling**	0.2816***	0.3322***	0.2721***	0.1917***	0.2129***	0.1820***
Controls	Y	Y	Y	Y	Y	Y
Intercept	−27.9396*	−17.8034*	−37.773	−20.2924	11.7153	4.7597
R2	0.3046	0.3062	0.2459	0.2713	0.2463	0.2687

Note: *p < 0.05 **p < 0.01 ***p < 0.001.

The control variables in the models include: the natural logarithm of annual family income, family structure, number of siblings, parenting styles, region, and quality of school. The coefficients for the control variables and fit statistics are available upon request.

### Multivariate Regression Results

Table 3 presents regression coefficients of parental education and their interaction terms with contextual characteristics while controlling for the covariates (see Model 2). The coefficients for the interaction terms among 3 + generation NH-whites are not statistically significant, which indicates that the influence of father and mother’s educational levels on child’s education does not vary based on environmental ethnic capital for non-Hispanic white men and women. For 3 + generation Hispanic men, the coefficient of the interaction term between percentage of ethnic population and father’s schooling is statistically significant (column 3:$\;\beta_{6}$ = -0.4231, *p* < 0.05). This indicates that the percentage of Hispanics in the county where the child resided when he/she was a teenager has a negative effect on father-son’s schooling transmission. In other words, educational attainment would be higher if a 3 + generation Hispanic male teenager resides in a place where the percentage of Hispanics is higher. For 2nd generation Hispanic men, the coefficient of the interaction between percentage of the college-educated population and parents’ schoolings are statistically significant (column 5: $\beta_{6\;of\;father}$ = −0.0079, *p* < 0.05; $\beta_{6\;of\;mother}$ = -0.0072, *p* < 0.05). The results suggest that the transmission of schooling from father and from mother vary according to the percentage of college-educated persons in the county where the child resided as a teenager. Educational attainment would be higher if the son of Hispanic immigrants resides in a county where the college-educated population is larger. In addition, percentage of families below poverty level and median household income do not have a significant relationship with parental educational level across groups. Hispanic women of 3+ and 2nd generations do not show significant coefficients of interaction terms.

**TABLE 3:** OLS Regression results of schooling years.

	NH-Whites_3+		Hispanics_3+		Hispanic_2nd	
	Men (1)	Women (2)	Men (3)	Women (4)	Men (5)	Women (6)
FATHER’s Schooling						
** Panel A: % Ethnic population**						
Father’s schooling	0.3178*	0.1597	0.3028***	0.2397**	0.2852***	0.2311***
FS* % ethnic population	−0.0147	0.0813	−0.4321*	−0.2004	−0.2957	−0.2339
** Panel B: % college educated population**						
Father’s schooling	0.3636***	0.2985***	0.2191*	0.0748	0.4068***	0.0166
FS* % college-educated population	−0.003	−0.0033	−0.0004	0.006	−0.0079*	0.0072
** Panel C: Ln(median HH income)**						
Father’s schooling	1.2998	1.5355	2.1638	−0.3072	0.7439	−0.6931
FS* Ln (median HH income)	−0.1919	−0.121	−0.1812	0.0471	−0.0474	0.0802
** Panel D: % family below poverty**						
Father’s schooling	0.2732***	0.2063***	0.1709*	0.2220	0.2415***	0.2164**
FS* % family below poverty	0.0035	0.0021	0.0031	−0.0028	−0.0015	−0.0037
MOTHER’s Schooling						
** Panel A: % Ethnic population**						
Mother’s schooling	0.1633	0.1971	0.2737***	0.1291*	0.2505***	0.1546*
MS* % ethnic population	0.137	0.1595	−0.0537	0.3042	−0.1901	0.0283
** Panel B: % college educated population**						
Mother’s schooling	0.3335***	0.4154***	0.1371	0.2462*	0.3854***	0.0117
MS* % college educated population	−0.0028	−0.0042	0.0062	−0.0021	−0.0072*	0.0074
** Panel C: Ln(median HH income)**						
Mother’s schooling	1.5247	1.4908	0.3228	2.5472	0.1449	−0.3822
MS* Ln (median HH income)	−0.1152	−0.1074	−0.0054	−0.2158	−0.1138	0.0516
** Panel D: % family below poverty**						
Mother’s schooling	0.2480***	0.3327***	0.2237**	0.0911	0.2231**	0.1533
MS* % family below poverty	0.0035	−0.0002	0.0030	0.0109	−0.0016	0.0026

Note: *p < 0.05 **p < 0.01 ***p < 0.001.

The control variables in the models include: the natural logarithm of annual family income, family structure, number of siblings, parenting styles, region, and quality of school. The coefficients for the control variables and fit statistics are available upon request.

To illustrate the estimated effects of OLS regression with interactions, we have plotted how estimated years of schooling of 3 + generation and 2nd generation Hispanic men would be expected to vary based on the percentage of the Hispanic population and the percentage of the college-educated population in the county. Estimates are shown for counties with a 0, 10, 20, 30, and 40 percent Hispanic population and college-educated population, combined with parent’s years of schooling of 1, 5, 10, and 15 years.

**FIGURE 1:**
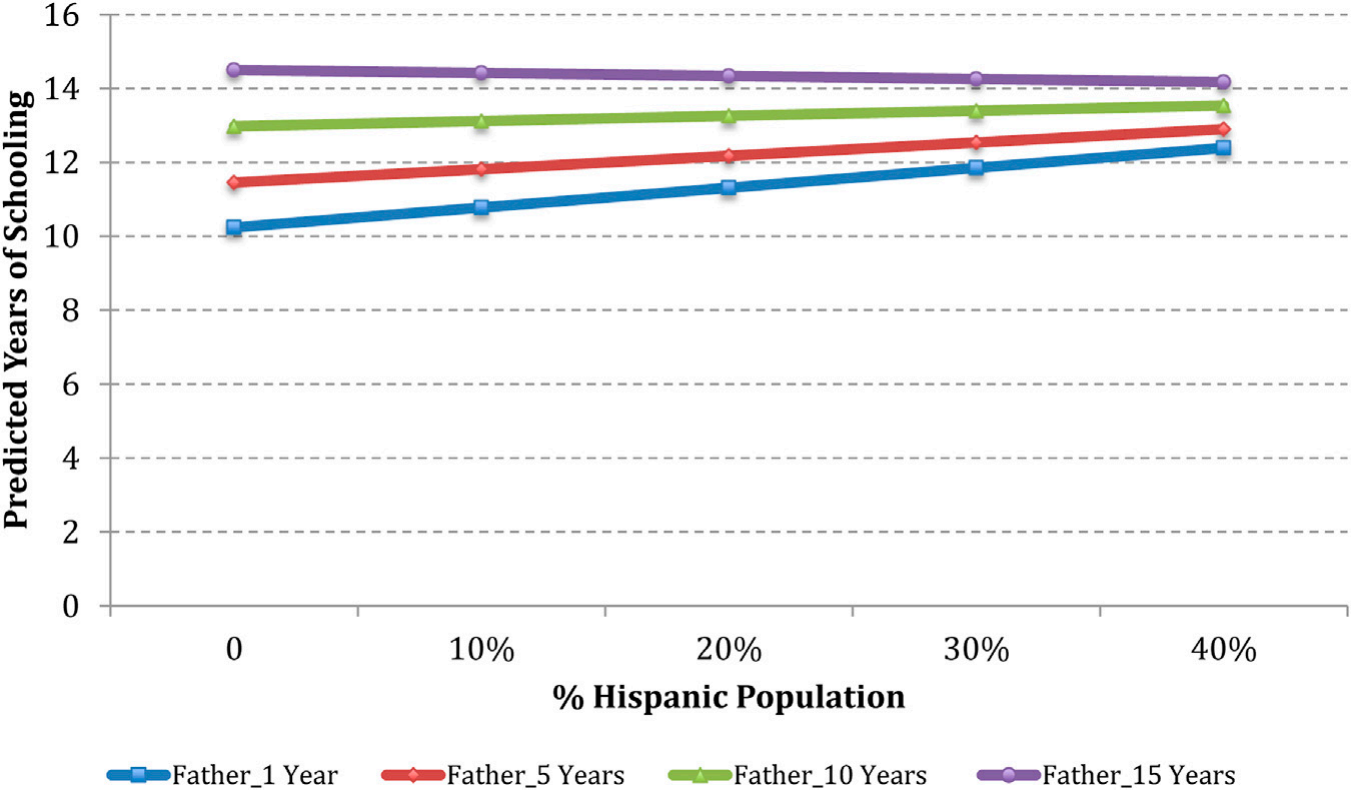
Predicted years of schooling of 3 + generation Hispanic men as a function of the level of father’s schooling and percentage of the Hispanic population in the county.

Based on the analyses in Table 3 column (3), Figure 1 presents predicted values of schooling years for a typical 3 + generation Hispanic man based on the percentage of the Hispanic population in the county. Using median values of control variables, a typical case is defined as a 3 + generation Hispanic man who has 1 sibling; whose mother gave birth to him when she was 25 years old; whose family income in 1996 was $41,513 after adjusting for inflation in 2010; who grew up with both biological parents in a non-South metropolitan area; and whose parents were authoritative, attended Teacher-Parent meetings, and volunteered at schools. In addition, this child felt safe at school, teachers were engaged in his education, and his education was not disrupted by other students in school.

Plots in Figure 1 reveal that if the father has 10 or 15 years of schooling, there is no discernible variation in estimated years of schooling for the child when there is an increase in the Hispanic population. However, if the father has 1 year of schooling, increasing the percentage of the Hispanic population in the county from 0 to 40 yields an estimated years of schooling increase from 10.2 to 12.4 years among 3 + generation Hispanic men. In addition, if the father has 5 years of schooling, increasing the percentage of the Hispanic population in the county from 0 to 40 yields an estimated years of schooling increase from 11.5 to 13 years among 3 + generation Hispanic men.

**FIGURE 2:**
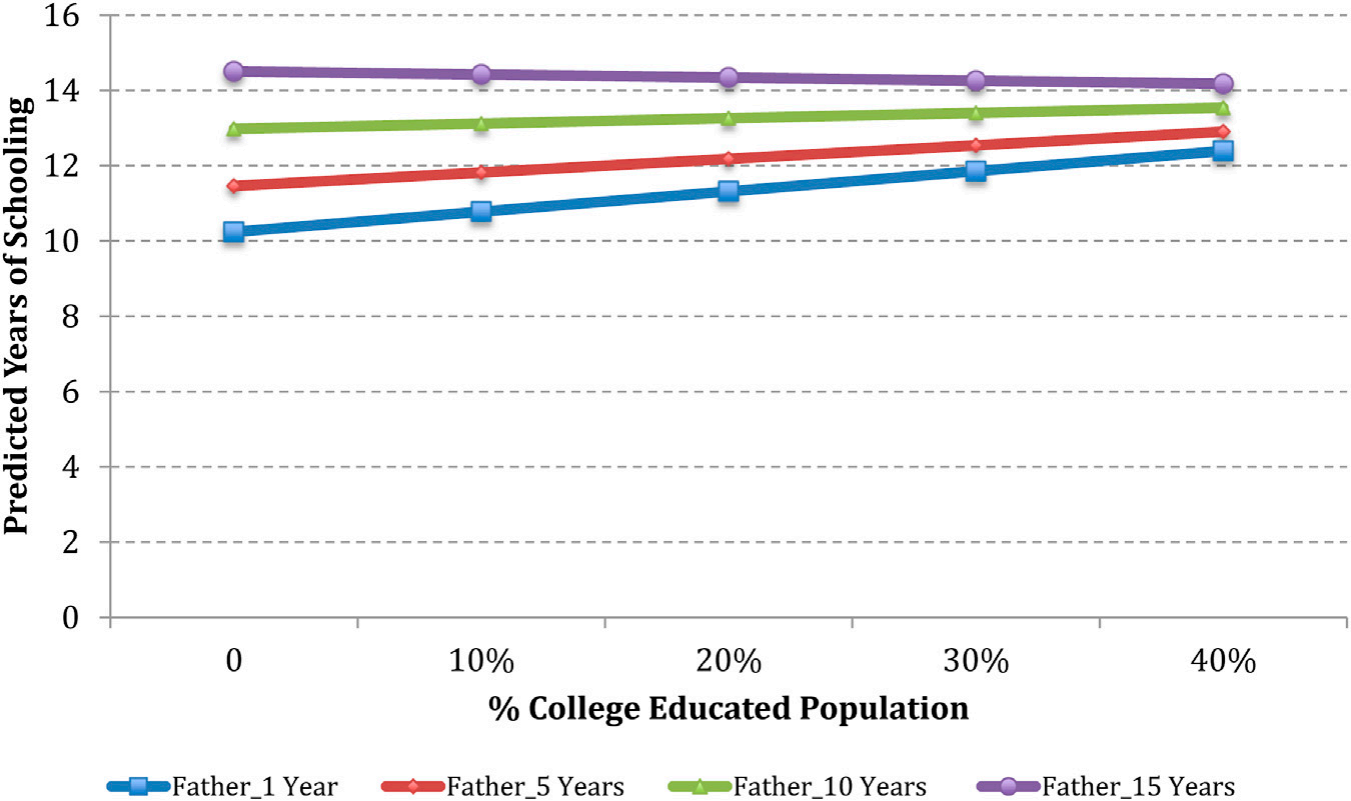
Predicted years of schooling of 2nd generation Hispanic men as a function of the level of father’s schooling and percentage of the college-educated population in the county.

**FIGURE 3:**
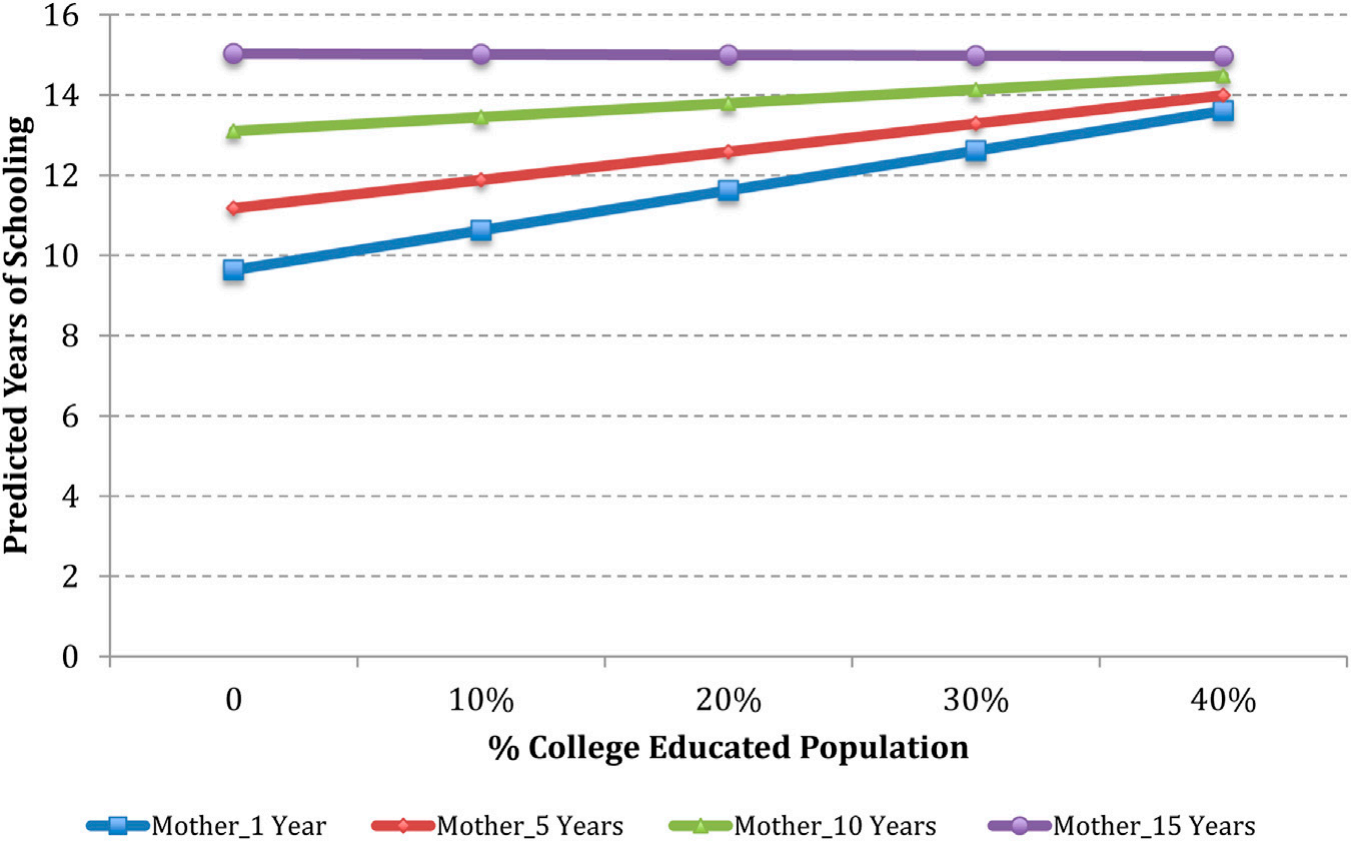
Predicted years of schooling of 2nd generation Hispanic men as a function of the level of mother’s schooling and percentage of the college-educated population in the county.

Based on the analyses in Table 3 column (5), Figure 2 and Figure 3 present predicted values of schooling years for a typical 2nd generation Hispanic man based on the percentage of the college-educated population in the county. Using median values of the control variables, a typical case is defined as a 2nd generation Hispanic man who has 1 sibling; whose mother gave birth to him when she was 25.5 years old; whose family income in 1996 was $30,443 after adjusting for inflation in 2010; who grew up with both biological parents in a non-South metropolitan area; and whose parents were authoritative, attended Teacher-Parent meetings, and volunteered at school. In addition, this child felt safe at school, teachers were engaged in his education, and his education was not disrupted by other students in school.

Data presented in Figure 2 indicate that for a typical 2nd generation Hispanic man, an increase in the college-educated population from 0 to 40 percent yields an increase in estimated years of schooling from 10.2 to 12.3 years if the father has an education of 1 year; an increase in estimated years of schooling from 11.5 to 13 years if the father has an education of 5 years; and an increase in estimated years of schooling from 13 to 13.5 if the father has an education of 10 years. However, there is no distinct variation of estimated years of schooling based on the percentage of college-educated population if the father has an education of 15 years.

Figure 3 presents estimated years of schooling of typical 2nd generation Hispanic men based on Mother’s years of schooling and percentage of the college-educated population in the county. An increase in the college-educated population from 0 to 40 percent yields an increase in estimated years of schooling of typical 2nd generation Hispanic men from 9.6 to 13.6 years if the mother has an education of 1 year; an increase in estimated years of schooling from 11.1 to 14 years if the mother has an education of 5 years; and an increase in estimated years of schooling from 13 to 14.5 if the mother has an education of 10 years. However, there is no distinct variation of estimated years of schooling based on the percentage of the college-educated population if the mother has an education of 15 years.

## Discussion and Conclusion

Hispanic Americans are an important demographic group with unique challenges. The present study examines whether the educational transmission from parents to children varies by county level demographic and socioeconomic characteristics among Hispanic children of immigrants and Hispanic children of native-born Americans. Men and women are analyzed separately. The benchmark population, 3 + generation non-Hispanic whites, is also included in the analysis as a reference group. While several studies have examined educational attainments among Hispanics (Wojtkiewicz and Donato, 1995; Chiswick and Noyna, 2004) and the importance of parental schooling in explaining group educational disparities (Kao and Tienda, 1995; Feliciano, 2005), little research has investigated how intergenerational transmission of education among Hispanics is affected by the social and demographic characteristics of the place where Hispanic children were raised. This topic is important given the well-documented significance of parental education and contextual characteristics in affecting the socioeconomic attainments of descendants of immigrants.

Our results indicate that contextual demographic socioeconomic characteristics do not seem to affect intergenerational transmission of education among NH-white men and women. Rather, their educational attainments seem to be affected largely by their parental educational level (see coefficients from Table 2 and Table 3). One possible interpretation of this result is that, among NH-whites, environmental characteristics, such as racial composition and poverty level, do not affect the transmission of parental human capital to children very much. However, contextual level characteristics seem to play a role in intergenerational transmission among Hispanic children. Our results confirm the importance of the interplay between contextual level characteristics and parental education on children’s attainment. Not all contextual level social or demographic characteristics have a significant compounding effect when paired with parental education. For example, regarding 3 + generation Hispanics, our findings suggest that having a higher percentage of Hispanic persons in the county where the Hispanic child resided as an adolescent decreases intergenerational persistence of education between father and son. In other words, educational attainment would be higher for 3 + Hispanic men residing in a county with a larger Hispanic population with their father’s education being constant. These demographic characteristics, however, do not affect 3 + generation Hispanic women. This result is not surprising given that Hispanic youths living in a high-density Hispanic community might benefit from sociocultural advantages, and these advantages might outweigh the disadvantages of the socioeconomic characteristics of the neighborhood (Eschbach et al., 2004). For example, minority children raised in an area with a higher percentage of minorities might face less discrimination and prejudice in public institutions, such as school. In addition, if teacher and minority students share a similar racial/ethnic background, students usually perform better in school (Dee, 2005; Bates and Glick, 2013) since a demographically similar teacher raises a student’s academic motivation and expectations (Steele, 1997). It is possible that 3 + generation men raised in areas with a higher percentage of Hispanics are more comfortable with the environment, are less likely to be stereotyped, experience less marginalization, and also have greater support and larger social networks. All these factors positively affect Hispanic men’s education.

However, it is surprising to see that the percentage of Hispanics in the county does not affect intergenerational educational mobility among Hispanic youths from immigrant families, as previous work has suggested that ethnic population in the neighborhood affects socioeconomic outcomes of children from immigrant families (Borjas and George, 1992). Rather, results demonstrate that the percentage of college-educated people in the county where the 2nd-generation Hispanic youth grew up has a negative effect on father-son and mother-son transmission of schooling. This suggests that a higher percentage of college-educated people in the county increases educational attainment among 2nd generation Hispanic men. Previous research has investigated the effects of residence characteristics and neighborhood quality on educational outcomes of children and youths (Patacchini and Zenou, 2011). However, these prior studies have focused primarily on the economic conditions of the neighborhood, such as poverty level and income level (Massey, 1996). Our findings regarding second-generation Hispanics provide novel insights into how educational level of the community may positively affect immigrant youths’ outcomes.

One limitation of this study is that the contextual level variables are measured at the county level, which might involve relatively larger units compared to census blocks. This limitation is in part due to data availability in the research area of intergenerational mobility (Sakamoto and Wang, 2020). Smaller geographic units, if available, may have potentially resulted in less bias for examining whether intergenerational educational mobility varies based on contextual level characteristics. However, the benefit to using county level variables, is that they have the capacity to yield results with greater policy implications. As many researchers have suggested, policies established at the county level show greater efficacy in the U.S. (Hoyman et al., 2016). In addition, the county level is small enough for many people to have some awareness of their co-residents in contrast to an entire metropolitan area or state.

Overall, the present findings have significant implications for public policy. As the Hispanic population continues to grow, issues surrounding socioeconomic status and assimilation level of Hispanic immigrants and their descendants have attracted public attention. Considering that Hispanic immigrants in general have lower educational and economic attainments compared to other groups, it is important for policymakers to provide Hispanic children with the means for upward mobility in order to increase equality. Children of Hispanics are usually disadvantaged in terms of parental socioeconomic support. However, my research suggests that children of Hispanics can benefit from certain environmental factors. If Hispanic children reside in an area with a large Hispanic population and a large college-educated population, they will most likely have higher educational mobility. Therefore, policymakers could focus on improving the socioeconomic quality of neighborhoods in order to provide 2nd-generation Hispanic children with an equal opportunity to succeed in their educational endeavors.

In terms of scientific implications, the present findings build on previous research which has emphasized the importance of parents’ human capital and ethnic environment in determining the educational attainments of children from immigrant families. The present study also provides novel insights regarding the interactive relationship between parental capital and ethnic environment and how this interaction influences different population groups categorized by race, immigration generation, and gender. Future research should be conducted to determine whether parental capital and contextual environment also interact to influence the educational attainments of other immigrant groups, such as Asian Americans. In addition, future research could investigate why intergenerational educational transmission is resilient against environmental factors among 2nd and 3 + generation Hispanic women.

## Data Availability

Some variables in the study are restricted. For those interested in the restricted NLSY data, please visit https://www.bls.gov/nls/geocodeapp.htm.

## Footnotes

1 One might argue that multilevel modeling is a more effective approach. However, the aim of this study was to investigate whether the relationship between children’s years of schooling and parental years of schooling depended on the characteristics of the county in which the child was raised. Therefore, cross-level interaction in OLS models is a more appropriate approach. Future studies focusing on testing whether parental education and contextual level characteristics were predictive of children’s schooling might make use of multilevel modeling.
